# A Novel Concentric Circular Coded Target, and Its Positioning and Identifying Method for Vision Measurement under Challenging Conditions

**DOI:** 10.3390/s21030855

**Published:** 2021-01-28

**Authors:** Yan Liu, Xin Su, Xiang Guo, Tao Suo, Qifeng Yu

**Affiliations:** 1 College of Physics and Optoelectronic Engineering, Shenzhen University, Shenzhen 518060, China; yan_liu@szu.edu.cn (Y.L.); yuqifeng58@139.com (Q.Y.); 2 Institute of Intelligent Optical Measurement and Detection, Shenzhen University, Shenzhen 518060, China; 3 School of Aeronautics, Northwestern Polytechnical University, Xi’an 710072, China; xin.su@mail.nwpu.edu.cn (X.S.); suotao@nwpu.edu.cn (T.S.); 4 International Research Laboratory of Impact Dynamics and Its Engineering Application, Xi’an 710072, China

**Keywords:** vision measurement, concentric circular coded targets, positioning and identifying method, flat viewing angle, eccentricity error

## Abstract

Coded targets have been demarcated as control points in various vision measurement tasks such as camera calibration, 3D reconstruction, pose estimation, etc. By employing coded targets, matching corresponding image points in multi images can be automatically realized which greatly improves the efficiency and accuracy of the measurement. Although the coded targets are well applied, particularly in the industrial vision system, the design of coded targets and its detection algorithms have encountered difficulties, especially under the conditions of poor illumination and flat viewing angle. This paper presents a novel concentric circular coded target (CCCT), and its positioning and identifying algorithms. The eccentricity error has been corrected based on a practical error-compensation model. Adaptive brightness adjustment has been employed to address the problems of poor illumination such as overexposure and underexposure. The robust recognition is realized by perspective correction based on four vertices of the background area in the CCCT local image. The simulation results indicate that the eccentricity errors of the larger and smaller circles at a large viewing angle of 70° are reduced by 95% and 77% after correction by the proposed method. The result of the wing deformation experiment demonstrates that the error of the vision method based on the corrected center is reduced by up to 18.54% compared with the vision method based on only the ellipse center when the wing is loaded with a weight of 6 kg. The proposed design is highly applicable, and its detection algorithms can achieve accurate positioning and robust identification even in challenging environments.

## 1. Introduction

Recently, vision measurement has become an extensively employed application, owing to the rapid advancement of image acquisition devices and computer vision techniques and its advantages of simple equipment, ease of use, low cost, strong environmental adaptability, fast, non-contact, and high accuracy [1,2,3,4]. The primary information including the position, shape, attitude, deformation, and dynamics of the objects can be derived from the vision measurement. Consequently, this method has become highly advanced with extensive application in various fields such as industry [5,6,7], civil infrastructure [8,9], food [10], medicine health [11,12], transport [13,14,15], security surveillance, etc. Particularly, the vision-based measurement techniques have gathered increasing attention in aerospace research and applications [16,17,18,19].

In vision measurement, extracting image points and matching the extracted points [20] in the images, from various sensors, or, gained by the same sensor in a different instance, are the keys and foundational steps. However, while performing, it results in an incorrect matching of feature points [21], besides being an extremely time-consuming task. To address these problems, methods of attaching artificial coded targets [22] have been developed. Coded targets are designed with a special “coded” symbol to produce unique identification. They can be automatically tracked and matched in multi-view or multi-time images, which significantly improve the efficiency and accuracy. Therefore, the artificial coded targets have been extensively used in vision measurements. Scaioni et al. [23] have used coded targets as ground control points to derive the calibration parameters. Liu and Dong et al. [24] have proposed multicamera systems to measure the displacements of large-span truss string structures. In their works, optical coded targets have been adhered to the structure to obtain the displacements at key points during the progressive collapse experiment. Huang [25] has performed the surface measurements of a mesh antenna by using coded targets. Liu et al. [18] have introduced coded targets in photogrammetric systems to successfully track the aerospace vehicles during a series of water impact tests and wind tunnel tests. Xiao [26] have used coded targets to perform the vision-based deformation measurement of steel structure beam of transmission tower foundation. Furthermore, Xiao et al. [27] have presented an accurate stereo vision system for industrial inspection, in which coded targets have been employed to calibrate vision systems and obtain a higher accuracy, conveniently.

**Figure 1:**
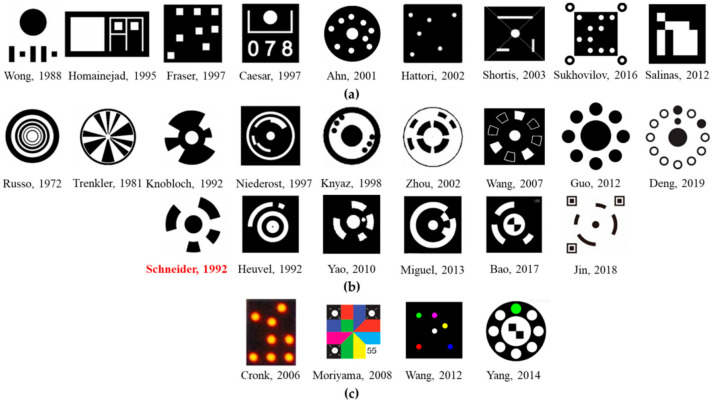
Various types of coded targets: (**a**) dot-dispersing form (**b**) centripetal form (**c**) color-coded targets.

According to the previous studies, vision measurements based on coded targets have been steadily increasing. Correspondingly, the requirements for accuracy have been increasing, thus various schemes of coded targets and their positioning and identification algorithms have been proposed in the publications to provide automatic detection, identification, and measurement. Figure 1 shows several common coded targets. They can be classified into two categories: dot-dispersing and centripetal categories. As shown in Figure 1a, a dot-dispersing form is that of a unique and identifiable pattern consisting of discrete circular or rectangle targets, or numerals [28,29,30,31,32,33,34,35]. Figure 1b shows the centripetal form, which consists of a center circle or diagonal target and a surrounding pattern similar to concentric rings with different widths [35], sector blocks with different areas [35], regularly arranged circles [36,37,38,39], or concentric ring segments [40,41,42,43,44,45,46,47,48,49,50]. Compared with the dot-dispersing form, centripetal form is more versatile and popular. Since the location of the centripetal coded target is based on the centroid of the central positioning circular or diagonal target, it enables fast and precise detection through ellipse fitting or least-squares matching. Moreover, the exclusive IDs of the coded targets have been embedded in the widths of concentric rings, the angles of concentric ring segments, or regularly arranged circles, which is prone to accurate identification. Besides, to take advantage of the color image information provided by the color digital cameras, color-coded targets [51,52,53] have been proposed for vision measurements, as shown in Figure 1c. However, effective color image information depends on the ambient light illumination and passive viewing targets. This is often a disadvantage for vision measurement, hence the studies on color-coded targets are scarce.

Amongst these code targets with centripetal pattern in Figure 1b, Schneider’s design is primarily applied in industrial vision measurements owing to the advantages of its simple structure, sufficient number of coding arrangements, and little impact of distortion on recognition. This kind of coded target is still widely in use since the 1990s. Many scholars have studied improving the design and locating the position and decoding. Wang et al. [42] have presented an improved encode method based on Schneider’s design and a new object recognition algorithm that combines with the geometrical feature of the new coded targets. Duan [43] has improved the decoding accuracy of the coded targets using a self-adaptive binary method. Liu et al. [44] have proposed an automatic and rapid algorithm for Schneider’s coded targets. Huang [45] have developed a method to detect accurately the center circles of coded targets based on certain criteria. Chen and Zhang [46] have proposed a model based on SIFT for recognizing the motion-blurred coded targets found in the moving objects. Miguel et al. [47,48] have presented a series of new coded targets and their identification and decoding algorithm, whose geometric structures are similar to Schneider’s design. Bao et al. [49] have developed a robust recognition and accurate locating method for circular coded diagonal target in which a diagonal target has been added in the central circle of Schneider’s design. Jin [50] has added three locators on the basis of coded target by Schneider to achieve precision position, besides increasing the number of targets.

**Figure 2:**
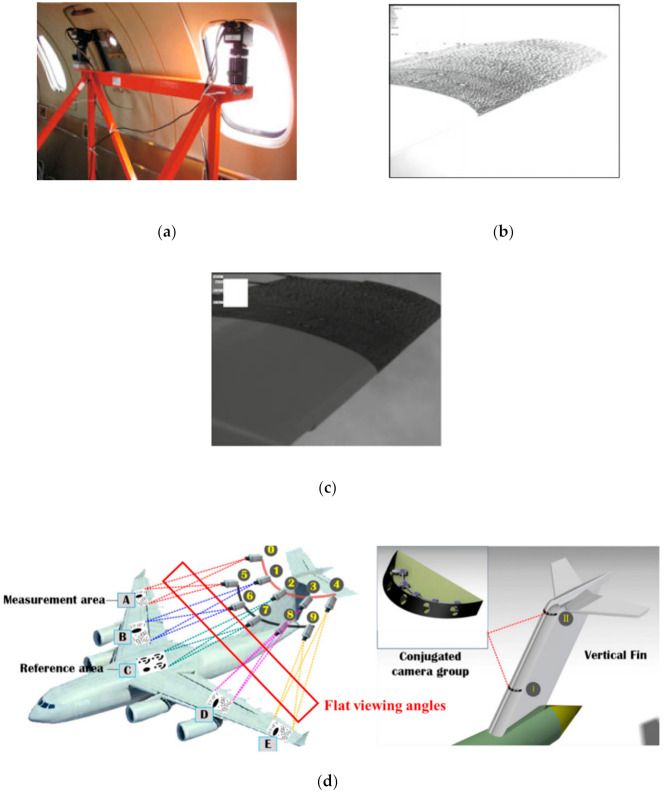
Vision system for an in-flight wing deformation measurement [54,55]: (**a**) the camera installed in the passenger compartment; (**b**) overexposure of the images owing to the changing illumination during the in-flight recording; (**c**) underexposure of the images; (**d**) the camera installed on the vertical fin for observing the wing speckle pattern and coded targets. Both of the two cases undergo flat viewing angles.

We note that most of the studies presented above have performed their tests only in laboratory conditions to check the performance of their coded targets’ design and detecting algorithms, which mandates a stable lighting environment and a favorable viewing angle. Once the vision method based on coded targets is applied in challenging conditions such as changing and non-uniform illumination, and the flat viewing angles, which were not involved in their works. The example in Figure 2 was taken from a real flight test for wing deformation measurement, where the images formed under overexposure (Figure 2b) and underexposure (Figure 2c) could often happen owing to significant light changes. These situations make it difficult to locate and identify a coded target.

**Figure 3:**
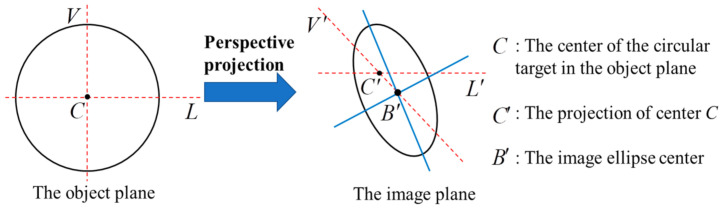
Eccentricity error of circular target caused by perspective imaging. *C* is the center of the circular target in the object plane; $C^{\prime }$ is the true projected center of the circular target; $B^{\prime }$ is the center of this image ellipse; the deviation between $C^{\prime }$ and $B^{\prime }$ is defined as eccentricity error.

Another critical issue, apart from the poor illumination problem, is the flat viewing angle, by which, the object being observed by the camera is apparently very flat. In the case of measuring the wing deformation, the installation locations of the image acquisition equipment, i.e., camera, are very limited and can only be on top of the fuselage or in the passenger compartment (Figure 2a [54]) or on the vertical fin of the aircraft (see Figure 2d [55]). Consequently, there is a tiny angle subtended between the plane of the coded target attached to the wing and the camera optical axis, and henceforth, the observation angle to the wing becomes flat. A flat viewing angle would cause severe distortion in the perspective images of the coded target. Strong perspective distortion may lead to both inaccurate positioning and low recognition, thereby decreasing the measurement accuracy, and even cause the failure of measurement. Moreover, the flat viewing angle also introduces a large eccentricity error [56,57], which indicates that the fitted ellipse center is inconsistent with the projected circle center of the coded target, as shown in Figure 3.

Hence, two major issues, i.e., the flat viewing angle and the harsh lighting conditions, hamper practical applications of vision measurements based on coded targets, which would be addressed in this investigation.

The main contributions of this study are as follows.

◼ We have designed a novel concentric circular coded target (CCCT) in which a concentric ring is employed to resolve the eccentricity error. This improvement aims at meeting the requirements of the flat viewing angle while measuring the important information (aero-elastic deformation, attitude, position, et al.) of objects such as wind tunnel models, flight vehicles, rotating blades, and other aerospace structures, in both ground and flight testing.
◼ We have proposed a positioning algorithm to precisely locate the true projected center of the CCCT. In this algorithm, the adaptive brightness adjustment has been used to address the problems of poor illumination. Concomitantly, the eccentricity error caused by flat viewing angle is corrected based on a practical error-compensation model.
◼ We have presented an approach to cope with the demands of the identification of CCCTs that make its use more robust in challenging environments, especially in the unfavorable illumination and flat viewing angle.

This paper is organized as follows: Section 2 performs a preceding test to estimate the applicability of Schneider’s coded targets in challenging conditions. Section 3 shows the design of proposed coded targets, and the methods to locate and identify our coded targets. Section 4 presents the simulation and real experiments to verify the proposed design and method in Section 4. Finally, Section 5 summarizes this study.

## 2. Preceding Tests for Estimating the Applicability of Schneider’s Coded Targets

**Figure 4:**
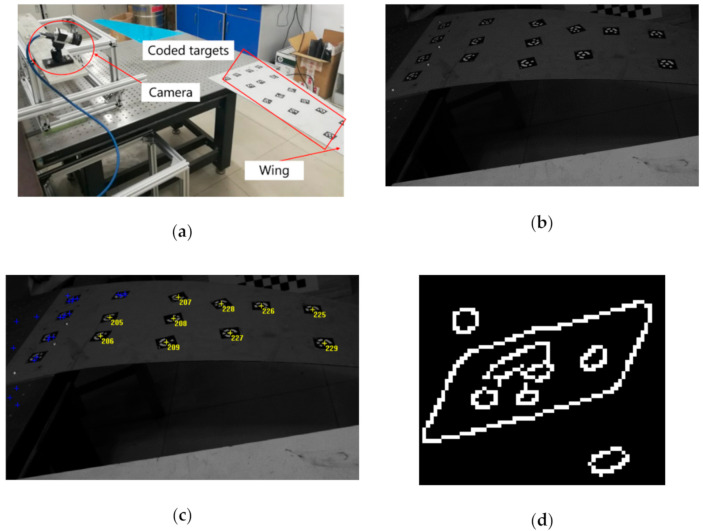
The experiment to verify the performance of Schneider’s design: (**a**) Experimental setup; (**b**) The real image has been taken with underexposure and heavily dark background; (**c**) The result of identifying the coded targets using commercial software; (**d**) The canny edge detection result of the coded target in the upper left corner.

To estimate the performance of Schneider’s design for vision measurement in challenging conditions, the case when the camera is installed on the vertical tail to measure the wing deformation has been simulated. As shown in Figure 4a, a digital camera (Basler acA1300-200 um with a global shutter) is installed on the rigid support links to acquire the images of aluminum alloy flat wing. Figure 4b shows an original image (1080 × 1024 resolution) including 15 coded targets attached to the wing surface, which has been taken with underexposure and heavily dark background. Accordingly, the viewing angle decreases with the closing to the top left, and the image condition becomes increasingly unfavorable.

Using commercial software (XJTUDP [13]), the identified coded targets are shown in Figure 4c. Five of the 15 coded targets which are far away from camera are not recognized. Figure 4d shows the result of the Canny edge detection for the coded target in the upper left corner. It suggests that the failure of identification could be directly attributed to the adhesion between the central positioning circle and the coded band. This phenomenon has been caused by strong perspective distortion under the flat viewing angle. Besides, it is in the locating step that poor illumination also presents a major challenge.

## 3. Methods

### 3.1. Design of the Concentric Circular Coded Target

To address the problem of decoding failure that results from poor illumination and flat viewing angle, we propose a 15-bits concentric circular coded target (CCCT) based on Schneider’s design. Further, we present its positioning and recognition method (see Section 3.2 and Section 3.3).

**Figure 5:**
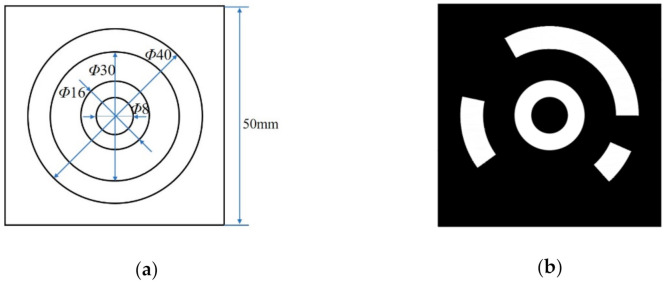
(**a**) Design scheme of the concentric circular coded target (CCCT); (**b**) One example of the CCCTs.

Figure 5 shows the design scheme of the CCCT. Each CCCT consists of two parts: a central positioning ring and the concentric circular coded band. The diameters of the central concentric circles are 8 and 16 mm respectively. The inner and outer circular edges of the coded band are 30 and 40 mm in diameters, respectively. The black square containing the CCCT is 50 mm in length. The above geometric parameters can be regarded as a set of basic proportions. In practical application, the users can enlarge or reduce the above parameters according to photographed distance. In [57], Liu et al. proposed a novel and practical method on size selection of circular coded targets to guarantee high-precision vision measurement.

The improvement mainly lies in two aspects. The initial step is to increase the ratio of the area between the central positioning circle and the circular coded band in CCCT, which aims at handling the problem of adhesion between the central positioning circle, and the circular coded band caused by flat viewing angle and poor illumination. First, we increase the ratio of the area between the central positioning circle and the circular coded band in CCCT. Second, we change the central positioning dot to a ring, which cannot correct the eccentricity error caused by the perspective distortion to improve the positioning accuracy but also achieves the perspective correction of CCCT local image to increase the recognition rate.

### 3.2. High-Precision Positioning of Concentric Circular Coded Targets

**Figure 6:**
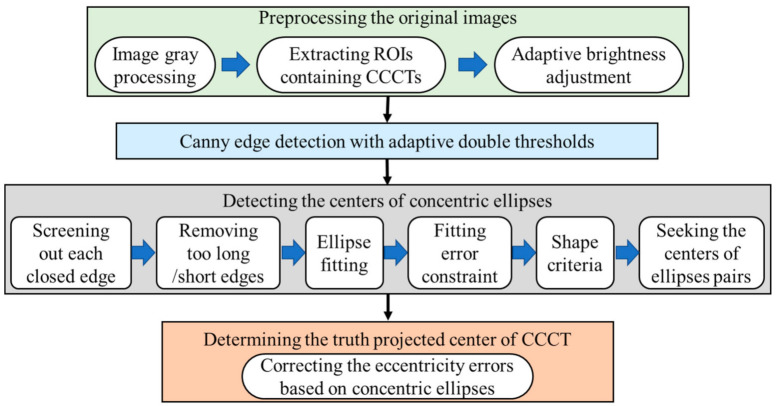
The workflow to locate the CCCTs.

The CCCTs should be able to be accurately located, as well as automatically identified. The position of a coded target must be located first to perform the identification then. Figure 6 schematically shows our workflow to locate the true projected center of the CCCT.

#### 3.2.1. Preprocessing the Original Images

Image preprocessing consists of three parts: graying, coarse localization to extract the regions of interest (ROIs), which is the local image containing each CCCT, and the adaptive brightness adjustment. First, it is necessary to convert the color image into a gray image if the original is a color image.

Thereafter, the extraction of ROIs that contains CCCTs is followed. We convert the gray image into a binary image, construct a rectangular area with proper length, and search the area with the high-intensity circle or ring. It is noted that the rectangular area should be set to contain each possible CCCT. Re-do searching is performed until all the areas are marked. For the continuous acquisition of images, the CCCT locations in the previous frame can be utilized to extract the ROIs of the current frame.

**Figure 7:**
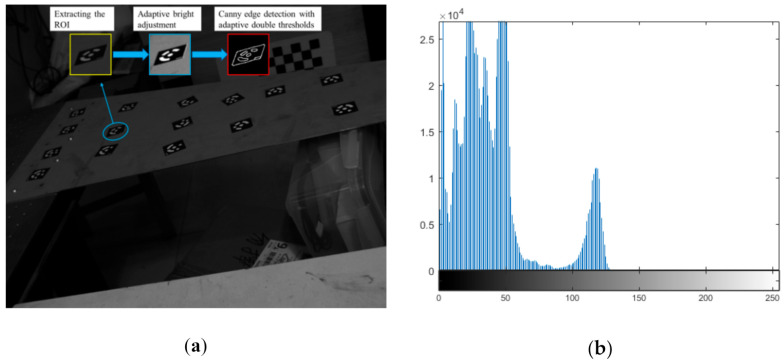
Result of adaptive Gamma correction: (**a**) Enhancing local image; (**b**) Histogram of original image.

If the gray histogram of the extracted ROI is concentrated in a narrow range, details of the image are not clear enough, and hence the contrast is low. For instance, when the middle peak part of the histogram is too far to the left or right, it implies that the local image is underexposed or overexposed. In this case, an adaptive Gamma correction algorithm [58] has been performed to adjust the brightness to enhance the information of the coded targets in the image. The Stretchlim function of Matlab has been employed to find, adaptively, a segmentation threshold vector that can be provided for Gamma correction. One underexposed image from the experiment of Section 2 is taken as an example, as shown in Figure 7a. The gray histogram is plotted in Figure 7b. From Figure 7b, it is obvious that the histogram skews to the left. For the CCCT at the second row and second column in Figure 7a, the inset with the light blue border shows that the result of adaptive Gamma correction brightness. It can be seen that the brightness and contrast of the local image have been enhanced, and visual effects have been improved.

#### 3.2.2. Canny Edge Detection with Adaptive Double Thresholds

In previous works, the majority of scholars have detected ellipses using the Canny method [59] with given double thresholds. The manual selection of thresholds may decrease the applicability of our methods. To meet the requirements under challenging lighting conditions, the Canny edge detection algorithm with adaptive double thresholds has been employed to extract the contour points in the local gray image. The high and low thresholds can be obtained with the following expressions:

(1) $$\left\{\begin{array}{l} TH=\frac{\sum_{x=1}^{m}\sum_{y=1}^{n}G\left(x,y\right)I\left(x,y\right)}{\sum_{x=1}^{m}\sum_{y=1}^{n}G\left(x,y\right)}\\ TL=TH/3 \end{array}\right.$$

where *TH* and *TL* are high and low thresholds of Canny edge detection, *m* and *n* the width and height of the image, and *I*(*x*, *y*) representing the gray value of pixel (*x*, *y*). Further, *G*(*x*, *y*) is defined as

(2) $$G(x,y)=\max\left\{\left|g_{x}\left(x,y\right)\right|,\left|g_{y}\left(x,y\right)\right|\right\}$$

where *g_x_*(*x*,*y*) and *g_y_*(*x*,*y*) are horizontal and vertical gradients of pixel (*x*, *y*), respectively. They can be calculated as

(3) $$g_{x}\left(x,y\right)=\left\{\begin{array}{l} I\left(x+1,y\right)-I\left(x,y\right),\;\;\;\;\;x=1\\ I\left(x,y\right)-I\left(x-1,y\right),\;\;\;\;\;x=m\\ \left(I\left(x+1,y\right)-I\left(x-1,y\right)\right)/2,\;\mathrm{others} \end{array}\right.$$

(4) $$g_{y}\left(x,y\right)=\left\{\begin{array}{l} I\left(x,y+1\right)-I\left(x,y\right),\;\;\;\;\;y=1\\ I\left(x,y\right)-I\left(x,y-1\right),\;\;\;\;\;y=n\\ \left(I\left(x,y+1\right)-I\left(x,y-1\right)\right)/2,\;\mathrm{others} \end{array}\right.$$

Therefore, the appropriate thresholds for the images in changing environments can be obtained automatically. Using a pair of thresholds *TH* and *TL*, the Canny edge detection algorithm has been used to extract strong edge pixels. In Figure 7a, the inset with red border shows the result of Canny edge detection with adaptive double thresholds.

#### 3.2.3. Detecting the Centers of Concentric Ellipse Pairs

◼ Screening out each closed edge

The central concentric ellipses would be imaged to closed contours. However, certain straight edges appear in the image after Canny edge detection. These straight lines might be generated by the noise or other objects in the background. Therefore, the possible edges of central circles that meet the following closed criteria should be screened out. Suppose that $A^{j}=\left\{P_{1}^{j},P_{2}^{j},\cdots ,P_{N}^{j}\right\}$ is the *j*’th extracted contour points, $P_{i}^{j}=\left(x_{i}^{j},y_{i}^{j}\right)$ is the *i*’th pixel on the *j*’th contour, and *N* is the number of pixels on this contour. If the end points of the edge satisfy the following formula in Equation (5), this edge is considered closed.

(5) $$\left|\bar{P_{1}^{j}P_{N}^{j}}\right|<T_{1}$$

The subscripts 1 and *N* denote the start and end points of the *j*’th contour. A typical value for *T*_1_ is 2 or 3 pixels [60].

◼ Removing excessively long (short) edges

According to our previous study on suitable size selection of artificial coded targets [57], the approximate sizes of the imaging ellipses of central circles could be determined in advance. They could not be too long or too short. The number *N*, of pixels on the closed edge, is taken as the length. If the edge whose length satisfies Equation (6), it might be regarded as a false edge and should be removed as per the condition given as

*N* < *T*_2_ or *N* > *T*_3_ (6)

The thresholds *T*_2_ and *T*_3_ represent the minimum and maximum lengths, respectively, which are known in advance from the given imaging conditions.

◼ Ellipse fitting

**Figure 8:**
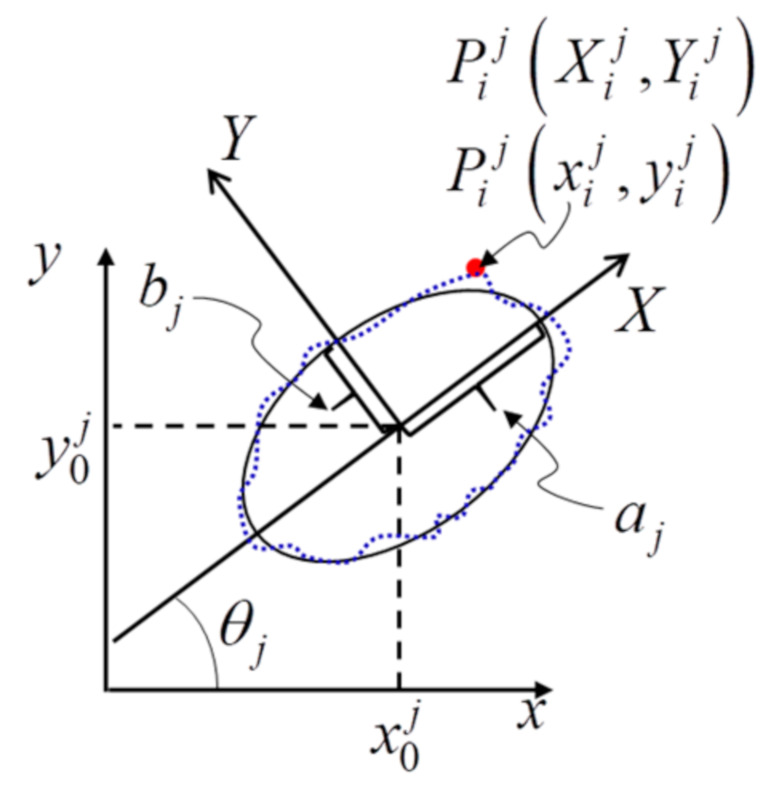
A fitting ellipse is located at $\left(x_{0}^{j},y_{0}^{j}\right)$ in the image coordinate system *xy*. The broken blue edge denotes the *j*’th closed edge in the image plane; $a_{j}$ and $b_{j}$ are semi-major axis and semi-minor axis, respectively; $\theta_{j}$ is the orientation angle; a temporary coordinate system *XY* at $\left(x_{0}^{j},y_{0}^{j}\right)$ is introduced to determine the ellipse fitting error for the *j*’th edge.

Subsequent to removing edges whose lengths do not satisfy the requirements, the bulk of the remaining edges might be the imaging ellipses of central circles. A least-squares ellipse fitting algorithm [45] has been adopted to obtain five ellipse parameters including the center coordinates $\left(x_{0}^{j},y_{0}^{j}\right)$ of *j*’th contour, semi-major axis $a_{j}$, semi-minor axis $b_{j}$, and orientation angle $\theta_{j}$, as shown in Figure 8.

◼ Fitting error constraint

While fitting the ellipse, certain closed edges that satisfy the length criteria might be the contours of objects in the background. However, they also have been fit to ellipses. Therefore, the fitting error constraint is applied to select the edges whose shapes are close to ellipses. In Figure 8, the broken blue edge denotes the *j*’th closed edge in the image plane, and (*x*, *y*) is the global image coordinate system. We introduce a temporary coordinate system *XY* at $\;\left(x_{0}^{j},y_{0}^{j}\right)$, and assume that the semi-major axis is *X* axis, and semi-minor axis is *Y* axis. For a given pixel $P_{i}^{j}\left(x_{i}^{j},y_{i}^{j}\right)$ on the *j*’th edge, the corresponding coordinates $\left(X_{i}^{j},Y_{i}^{j}\right)$ in *XY* coordinate system can be obtained as

(7) $$\left\{\begin{array}{l} X_{i}^{j}=\left(x_{i}^{j}-x_{0}^{j}\right)\cos\theta_{j}+\left(y_{i}^{j}-y_{0}^{j}\right)\sin\theta_{j}\\ Y_{i}^{j}=\left(x_{i}^{j}-x_{0}^{j}\right)\sin\theta_{j}+\left(y_{i}^{j}-y_{0}^{j}\right)\cos\theta_{j} \end{array}\right.$$

Therefore, the ellipse fitting error for the *j*’th edge is computed as

(8) $$\Delta r^{j}=\sqrt{\frac{1}{N-1}{\sum_{i=1}^{N}\left(\sqrt{{\left(X_{i}^{j}\right)}^{2}+{\left(Y_{i}^{j}\right)}^{2}}-\frac{a_{j}b_{j}\sqrt{{\left(X_{i}^{j}\right)}^{2}+{\left(Y_{i}^{j}\right)}^{2}}}{\sqrt{{\left(a_{j}Y_{i}^{j}\right)}^{2}+{\left(b_{j}X_{i}^{j}\right)}^{2}}}\right)}^{2}}$$

The edge whose $\Delta r_{i}^{j}$> *T*_4_ is considered as an undemand contour, and it should be deleted. A typical value of *T*_4_ is 1 pixel.

◼ Shape criteria

The rate of semi-minor axis $b_{j}$ to semi-major axis $a_{j}$ has a maximum value of 1 for a circular contour, and decreases with the decreasing viewing angle. According to Ahn’s study [45], the *form factor* is defined as

(9) $$form\;factor=\frac{{\left(perimeter\right)}^{2}}{4\pi \times area}$$

At the observe angle of 70°, the *form factor* has a value up to 1.5. The perimeter and area of an ellipse can be expressed by

(10) $$\left\{\begin{array}{l} area=\pi a_{j}b_{j}\\ perimeter=2\pi b_{j}+4\left(a_{j}-b_{j}\right) \end{array}\right.$$

Combining Equation (9) with Equation (10), the rate of semi-minor axis $b_{j}$ to semi-major axis $a_{j}$ should satisfy

(11) $$T_{5}\leq \frac{b_{j}}{a_{j}}\leq 1$$

where *T*_5_ is set to 0.41 at the viewing angle of 70°.

◼ Seeking the centers of ellipse pairs

For our proposed CCCT, a pair of ellipses is the projection of central concentric circles onto the image plane. Although the two ellipses have their separate centers in one image, the distance between the two centers is not too far. According to this characteristic, a threshold *T*_6_ could be set for the distance between the centers of inner and outer ellipses. If the centers of the ellipse pairs meet the following formula in Equation (12), they are considered as candidate contours of ellipse pair produced by coded targets and should be withheld.

The superscripts *in* and *out* denotes the inner and outer ellipses, respectively.

(12) $${\left[{\left(x_{0}^{in}-x_{0}^{out}\right)}^{2}+{\left(y_{0}^{in}-y_{0}^{out}\right)}^{2}\right]}^{1/2}\leq T_{6}$$

#### 3.2.4. Determining the Truth Projected Center of CCCT

**Figure 9:**
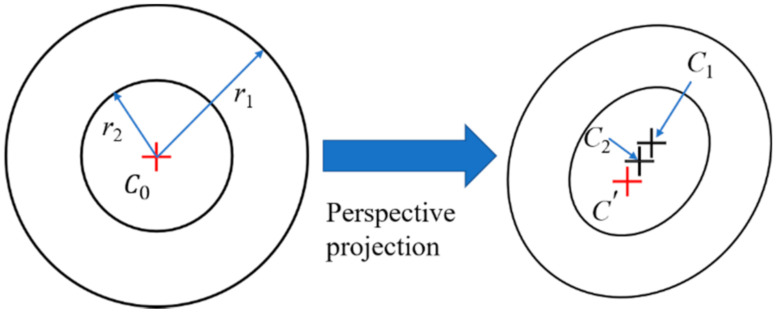
Perspective projection of central positioning ring of the coded target.

To obtain the true projected centers of the CCCTs, the two centers of each of the ellipse pair are used to correct the eccentricity errors. As shown in Figure 9, the central ring of a CCCT appears as two ellipses separated by two centers in the perspective image. Suppose that *C*_0_ is the center of CCCT in object space; *r*_1_ and *r*_2_ are the radii of outer and inner circles; *C’* is the image point of *C*_0_; *C*_1_ and *C*_2_ are the centers of outer and inner ellipses, respectively. From the works of Ahn [56] and He [61], the eccentricity errors of the outer and inner ellipses could be described as

(13) $$\left\{\begin{array}{l} \varepsilon_{x_{1}}=x_{C_{1}}-x_{C’}=K_{x}r_{1}^{2}\\ \varepsilon_{y_{1}}=y_{C_{1}}-y_{C’}=K_{y}r_{1}^{2} \end{array}\right.$$

(14) $$\left\{\begin{array}{l} \varepsilon_{x_{2}}=x_{C_{2}}-x_{C’}=K_{x}r_{2}^{2}\\ \varepsilon_{y_{2}}=y_{C_{2}}-y_{C’}=K_{y}r_{2}^{2} \end{array}\right.$$

where *K_x_* and *K_y_* are two coefficients independent of the radii *r*_1_ and *r*_2_, and are equal for two circles in central positioning ring of CCCT.

Combining Equations (13) and (14), the true projected center of the CCCT can be obtained as

(15) $$\left\{\begin{array}{l} x_{C’}=\frac{{\left(r_{1}/r_{2}\right)}^{2}x_{C_{2}}-x_{C_{1}}}{{\left(r_{1}/r_{2}\right)}^{2}-1}\\ y_{C’}=\frac{{\left(r_{1}/r_{2}\right)}^{2}y_{C_{2}}-y_{C_{1}}}{{\left(r_{1}/r_{2}\right)}^{2}-1} \end{array}\right.$$

where $\left(x_{C_{1}},y_{C_{1}}\right)$ and $\left(x_{C_{2}},y_{C_{2}}\right)$ are the center coordinates of the outer and inner ellipses.

### 3.3. Identification of Concentric Circular Coded Targets

**Figure 10:**
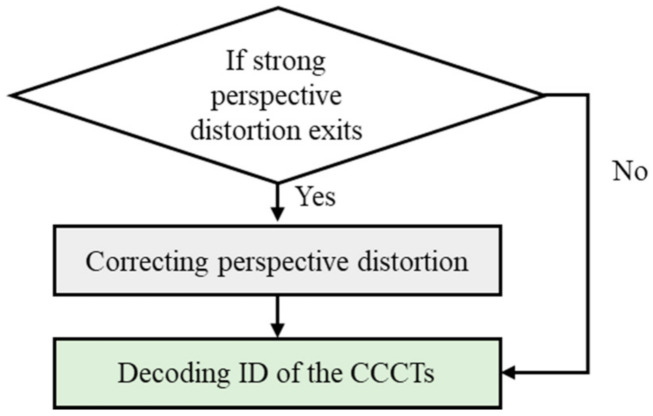
The workflow process to identify the number of CCCT.

Subsequent to accurate locating of the centers of CCCTs, this part focuses on identifying the associated number of each CCCT. In Figure 4, the identified results indicate that serious perspective distortion occurs at the flat viewing angle, besides being more difficult in identifying the coded targets. In this case, it is necessary to correct perspective distortion, with an alternative of direct identification of CCCTs. The workflow is given in Figure 10.

#### 3.3.1. Correcting Perspective Distortion

**Figure 11:**
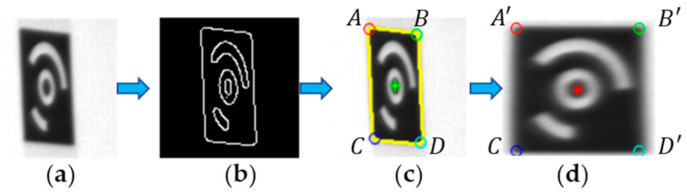
(**a**) The raw local image (ROI); (**b**) The result of Canny edge detection with adaptive double thresholds; (**c**) The result of determining the truth projected center (the green ‘*’) and the four vertices (the color ‘O’) of background area; (**d**) The result of correcting perspective distortion.

For the previously obtained center coordinates of candidate CCCTs, perspective correction is implemented for the ROIs covering them if no CCCT is identified in this region. First, the four vertices of the background area of the CCCT are determined. In this step, the Canny edge detection is performed over the local CCCT image shown in Figure 11a to get all the contours shown in Figure 11b. Thereafter, each of the detected contours is extracted and analyzed to see whether it is closed and comply with the size constraint. For a closed and large contour, for example, the yellow edge in Figure 11c, Gaussian convolution is performed to smooth the contour. Subsequently, a corner detection method based on global and local curvature properties [62] has been employed to obtain the four true vertices (see the color ‘O’).

Perspective distortion correction is an inverse process over perspective imaging. The basic step is to obtain perspective transformation matrix. Perspective transformation can be expressed as

(16) $$\left\{\begin{array}{c} x=\frac{a_{11}u+a_{12}v+a_{13}}{a_{31}u+a_{32}v+a_{33}}\\ y=\frac{a_{21}u+a_{22}v+a_{23}}{a_{31}u+a_{32}v+a_{33}} \end{array}\right.$$

In Equation (16), $\left(u,v\right)$ and $\left(x,y\right)$ are the points in the raw local image and corrected image, respectively. Further, $TP=\left[\begin{array}{ccc} a_{11} & a_{12} & a_{13}\\ a_{21} & a_{22} & a_{23}\\ a_{31} & a_{32} & a_{33} \end{array}\right]$ is the perspective transformation matrix. If *a*_33_ = 1, Equation (16) becomes

(17) $$\left\{\begin{array}{c} a_{11}u+a_{12}v+a_{13}-a_{31}ux-a_{32}vx=x\\ a_{21}u+a_{22}v+a_{23}-a_{31}uy-a_{32}vy=y \end{array}\right.$$

In Figure 11c, *A*$\left(u_{tl},v_{tl}\right)$, *B*$\left(u_{tr},v_{tr}\right)$, *C*$\left(u_{bl},v_{bl}\right)$, and *D*$\left(u_{br},v_{br}\right)$ represent the four vertices (top-left, top-right, bottom-left, and bottom-right) of the black background area in the raw local image. Subsequent to perspective rectification, we set the side length of the background area as $w=\min\left\{\sqrt{{\left(u_{br}-u_{tl}\right)}^{2}+{\left(v_{br}-v_{tl}\right)}^{2}},\sqrt{{\left(u_{bl}-u_{tr}\right)}^{2}+{\left(v_{bl}-v_{tr}\right)}^{2}}\right\}$. Then, $A^{\prime }\left(u_{tl},v_{tl}\right)$, $B^{\prime }\left(u_{tl}+w,v_{tl}\right)$, $C^{\prime }\left(u_{tl},v_{tl}+w\right)$, and $D^{\prime }\left(u_{tl}+w,v_{tl}+w\right)$ are the four vertices in the corrected image. Substituting the four pairs of image coordinates into Equation (5), ***TP*** can be solved by the following equations:

(18) $$\begin{array}{c} \begin{array}{c} \begin{array}{c} \left[\begin{array}{cccccccc} u_{tl} & v_{tl} & 1 & 0 & 0 & 0 & -u_{tl}u_{tl} & -v_{tl}u_{tl}\\ 0 & 0 & 0 & u_{tl} & v_{tl} & 1 & -u_{tl}v_{tl} & -v_{tl}v_{tl}\\ u_{tr} & v_{tr} & 1 & 0 & 0 & 0 & -u_{tr}\left(u_{tl}+w\right) & -v_{tr}\left(u_{tl}+w\right)\\ 0 & 0 & 0 & u_{tr} & v_{tr} & 1 & -u_{tr}v_{tl} & -v_{tr}v_{tl}\\ u_{bl} & v_{bl} & 1 & 0 & 0 & 0 & -u_{bl}u_{tl} & -v_{bl}u_{tl}\\ 0 & 0 & 0 & u_{bl} & v_{bl} & 1 & -u_{bl}\left(v_{tl}+w\right) & -v_{bl}\left(v_{tl}+w\right)\\ u_{br} & v_{br} & 1 & 0 & 0 & 0 & -u_{br}\left(u_{tl}+w\right) & -v_{br}\left(u_{tl}+w\right)\\ 0 & 0 & 0 & u_{br} & v_{br} & 1 & -u_{br}\left(v_{tl}+w\right) & -v_{br}\left(v_{tl}+w\right) \end{array}\right]\left[\begin{array}{c} a_{11}\\ a_{12}\\ a_{13}\\ a_{21}\\ \begin{array}{cc} \; & a_{22}\\ \; & a_{23}\\ \; & a_{31}\\ \; & a_{32} \end{array} \end{array}\right]=\left[\begin{array}{c} u_{tl}\\ v_{tl}\\ u_{tl}+w\\ v_{tl}\\ u_{tl}\\ v_{tl}+w\\ u_{tl}+w\\ v_{tl}+w \end{array}\right] \end{array} \end{array} \end{array}$$

Base on the perspective transformation matrix ***TP***, each pixel of the raw local image in Figure 11a can be mapped to the corrected image through Equation (16). However, this may be a one-to-one mapping or many-to-one mapping, and the mapped pixels (*x*, *y*) may not be integers, hence the bilinear interpolation is employed to calculate the gray value of each pixel in the corrected image. Figure 11d displays the result of perspective correction in Figure 11a, as it verifies the effectiveness of the perspective correction method in this paper.

#### 3.3.2. Identifying the ID of Each CCCT

Identifying the ID of each CCCT can be performed over the extracted ROI or its corrected local image at a flat viewing angle. The detailed process is as follows:

◼ First, scan the outward from the center of the CCCT to search the outer and inner boundaries of the circular coded band. The Otsu algorithm is employed to calculate the average gray of the pixels contained in the outer ellipse of the coded band, Further, this average value is taken as a threshold to distinguish the black background and white target.
◼ Then, for 15-bits CCCTs, rays are radiated outward from the center with an angular step of 2.4°. All pixels on each ray that fall between the outer and inner boundaries are sampled. The median gray value of these pixels is compared with the gray threshold obtained previously, and if it is greater than the threshold, the ray gray sample value is marked as “1.” Otherwise it is “0.” A 150-bits binary sequence can be obtained by scanning one circle clockwise or anticlockwise.
◼ The ideal starting point to decode is from the place where the gray level fluctuates sharply to avoid the small section in the coded band being erroneously identified. Therefore, we reverse the 150-bits binary sequence until the head and tail are not the same. The reordered sequence is divided into several blocks according to consecutive “1” or “0”. The number of “1” or “0” contained in the *k*’th block is defined as ***P***[*k*], and the “1” or “0” information of the block is placed in the array ***C***[*k*].
◼ Since the 15-bits binary code is adopted for the proposed CCCT, every 10 of 150 bits represent a binary bit, which is determined as

(19) $$B\left[k\right]=round\left(\frac{P\left[k\right]}{10}\right)$$

The binary bits obtained from all coded blocks form a 15-bits binary number. For example, the CCCT shown in Figure 11 has 6 blocks, viz., ***P*** = {60, 30, 10, 9, 10, 31}, ***C*** = {1, 0, 1, 0, 1, 0}, ***B*** = {6, 3, 1, 1, 1, 3}, and hence the 15-bits binary number is 111111000101000.

◼ Finally, the 15-bits binary number is ordered, the corresponding decimal number is computed, and the minimum value is used as the ID of CCCT.

## 4. Experiments and Results

To verify the accuracy and effectiveness of the proposed CCCT scheme and its positioning and identifying algorithm, the simulation and real experiments have been conducted in this work.

### 4.1. Positioning Accuracy Verification Experiment

#### 4.1.1. Influence of Different Viewing Angles

**Figure 12:**
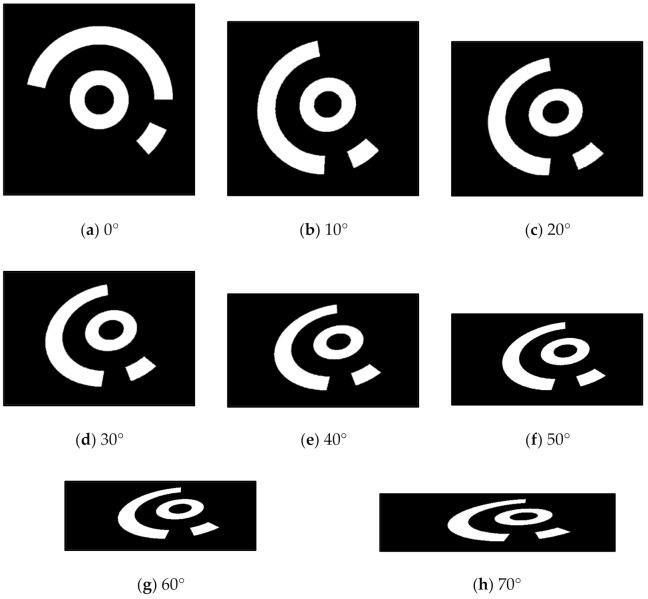
Simulation images with different viewing angles: (**a**) 0°; (**b**) 10°; (**c**) 20°; (**d**) 30°; (**e**) 40°; (**f**) 50°; (**g**) 60°; (**h**) 70°.

**Figure 13:**
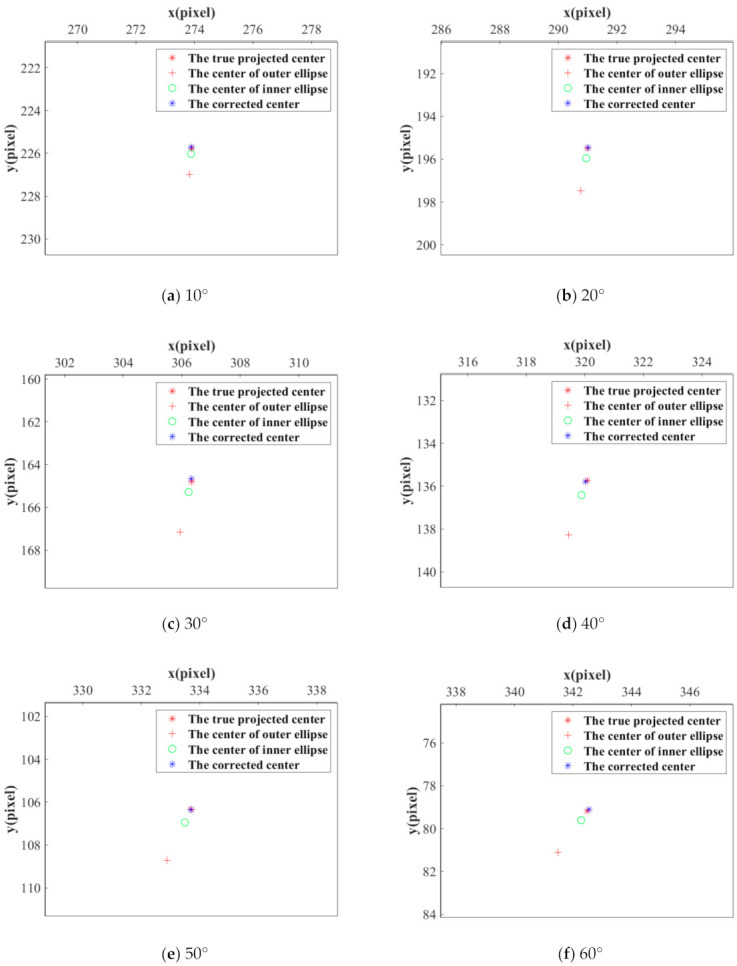

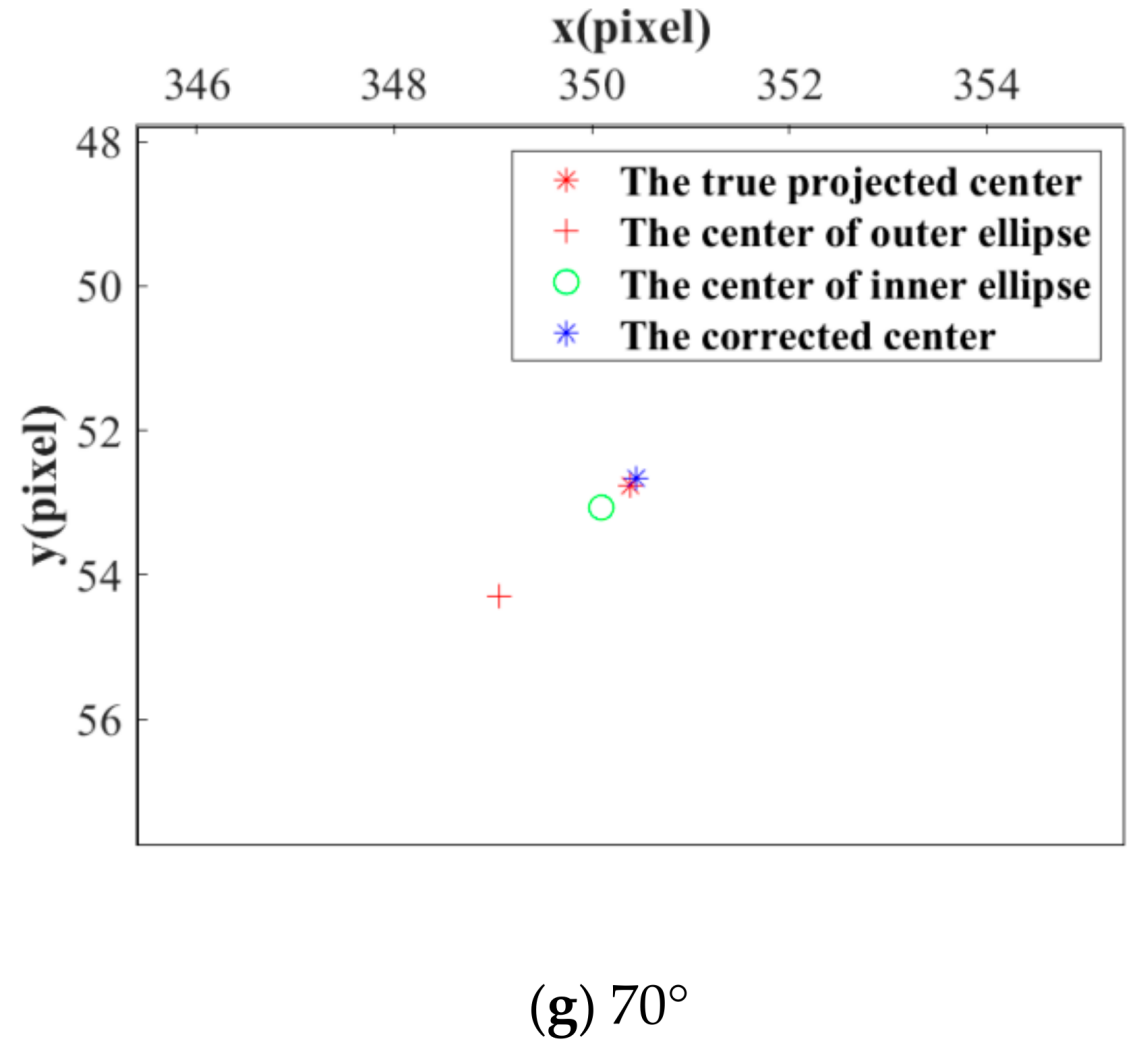
The center positioning results for different viewing angles: (**a**) 10°; (**b**) 20°; (**c**) 30°; (**d**) 40°; (**e**) 50°; (**f**) 60°; (**g**) 70°. The red “+” and green “o” represent the centers of the outer and inner ellipses detected by fitting method; the blue ‘*’ represents the CCCT corrected center obtained by our method; the red ‘*’ is the location of the true projected center.

**Figure 14:**
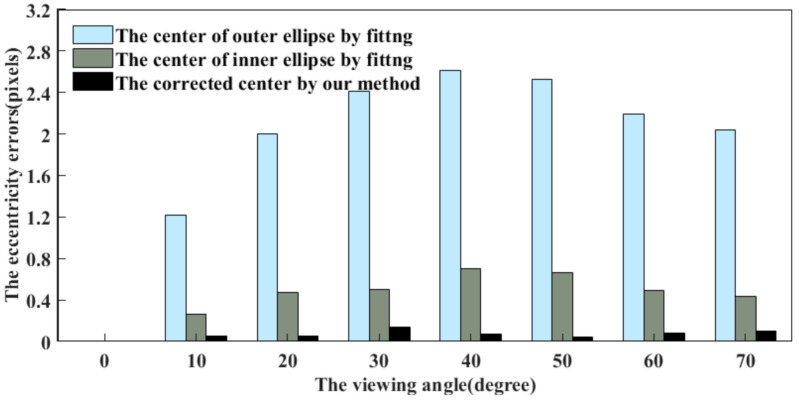
For different viewing angles, the eccentricity errors of the centers of the outer and inner ellipses using the fitting method in comparison with that of the CCCT corrected center by our method.

First, a front-view image of No.6 CCCT has been established by the simulated camera, as shown in Figure 12a, i.e., the viewing angle between the camera optical axis and the CCCT plane normal is 0°. The resolution is 512 × 512 pixels, and the coordinates of the centers of the two concentric circles are given as (257, 257) pixels. In Figure 12b–h, the viewing angle is set from 10° to 70° with an interval of 10°. For each image, the conventional ellipse fitting method is employed to obtain the centers of the outer and inner ellipses of the central positioning ring. The proposed method is used to compensate the eccentricity error. Figure 13 shows the center positioning results. The red ‘+’ and green ‘o’ represent the centers of the outer and inner ellipses; the blue ‘*’ represents the CCCT center obtained by our method, and the red ‘*’ is the location of the true projected center. The results show that the corrected center by our method is closer to the true projected center of each CCCT. To demonstrate the correction performance more clearly, a statistical analysis of the eccentricity errors is carried out, as shown in Figure 14. We note that both the centers of the outer and inner ellipses obtained by ellipse fitting method have a large eccentricity error, which increases significantly with the radius of the circle. Subsequent to correcting the eccentricity errors using our method, the eccentricity errors keep a very low level. At a large viewing angle of 70°, the eccentricity errors of larger and smaller ellipses reach 2.04 and 0.44 pixels while the eccentricity error by our method is 0.10 pixels. The eccentricity error is reduced by 95% and 77%. Therefore, the eccentricity error has been greatly decreased by positioning the center of the CCCT with our method.

#### 4.1.2. Influence of Front-View Image Resolution

**Figure 15:**
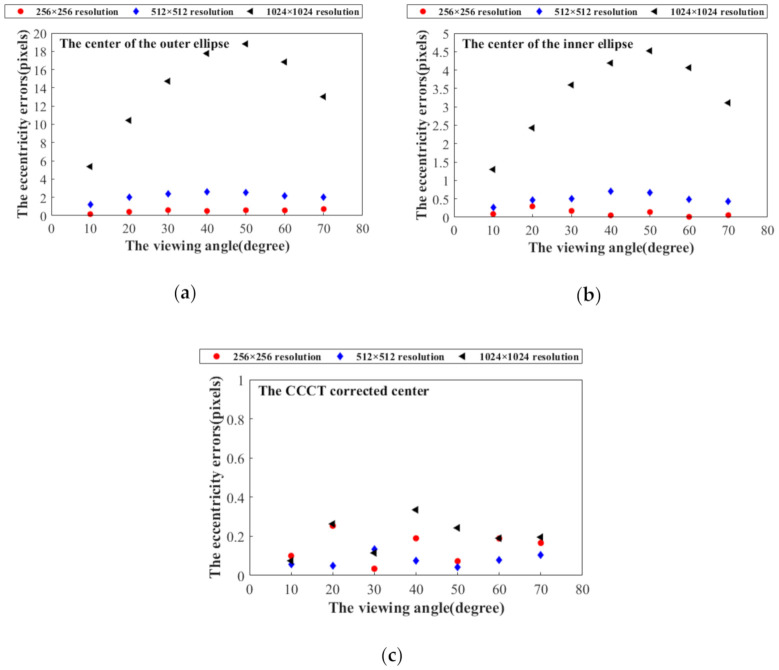
(**a**,**b**): The eccentricity errors of the outer and inner ellipses’ centers obtained by fitting method; (**c**) The results after correction by our method 10°.

The purpose of this subsection is to study how the CCCT detection algorithm is affected if the front-view image resolution is increased or decreased. Besides the series of images based on 512 × 512 resolution in Figure 12, we also adopt the simulated camera to produce the front-view images with 256 × 256 and 1024 × 1024 resolutions, respectively. Figure 15a,b shows the eccentricity errors of the centers of the outer and inner ellipses, obtained by the fitting method. Further, the results that are subjected to the correction by our method are shown in Figure 15c. By employing the ellipse fitting method, the eccentricity errors are enlarged with the increase of image resolution. Subsequent to the correction by our method, the eccentricity errors are smaller than 0.4 pixels under the influence of image resolution. Therefore, the front-view image resolution has little or no impact on the CCCT detection algorithm.

#### 4.1.3. The Performance against the Image Noises

To validate the performance against the image noises, we add a Gaussian noise and pepper noise to the simulated image of 30° in Figure 13d. The variance of the Gaussian noise, besides the density of pepper noise, is set from 0.005 to 0.02. Table 1 shows the eccentricity errors (EEs) under different image noises. The results suggest that the eccentricity error of the outer ellipse center obtained by fitting is five times larger than that of the inner ellipse center, and twenty times that of the CCCT corrected center. The EEs experience negligible change when the image noise is increased. Therefore, our positioning method is more accurate even under the condition of noise with varying intensities.

### 4.2. Identification Performance Verification Experiment

**Figure 16:**
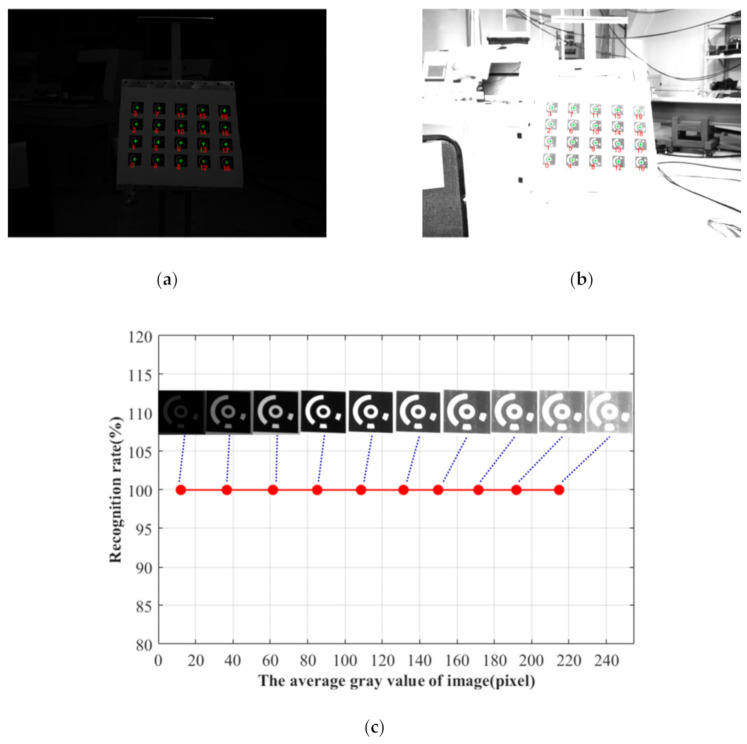
Experimental results of CCCT recognition under varying exposure levels: (**a**) Overexposed image; (**b**) Underexposed image; (**c**) The recognition rate for different exposure levels.

To check the proposed identifying method, the experiments under different lighting conditions and viewing angles have been conducted. The recognition rate of all the CCCTs in the field of view is regarded as a criterion for evaluating the recognition performance. A Daheng digital camera (DAHENG_MER-1070-14U3x) with a rolling shutter has been utilized to capture the images with the resolution of 3840 × 2748 pixels. It is noted that the following images are all taken when neither the camera nor the imaged object is moved, so the quality of the recorded coded targets is not affected by a rolling shutter effect. Each image contains 20 coded targets. By adjusting the brightness of the LED light source, ten images with varying exposure levels are acquired. Figure 16a,b shows under- and overexposed examples. In Figure 16, the green “+” represent the centers of the CCCTs by the presented positioning method, and the numbers in red represent their ID obtained by our identification method. Taking as an example the CCCT with ID 9, the average gray value of its ROI is calculated to estimate the exposure level. As shown in Figure 16c, the recognition rate for all the 20 coded targets is still 100% when the average gray value is varying from 12 to 215 pixels. It can be seen that our method can identify all the coded targets even when the illumination condition is poor (e.g., overexposure, underexposure).

**Figure 17:**
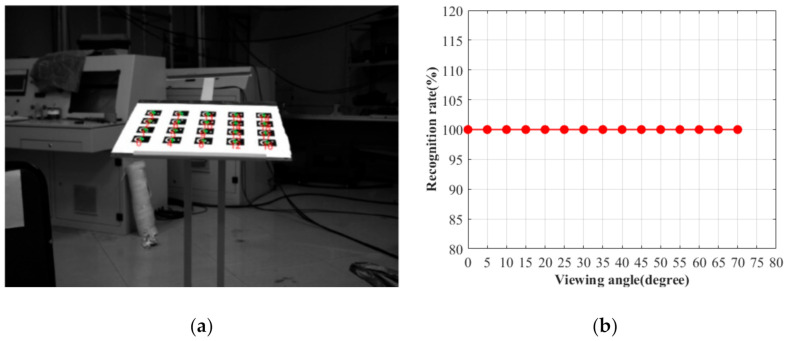
Experimental results of CCCT recognition under different viewing angles. (**a**) The viewing angle of 70°; (**b**) The recognition rate for different viewing angles.

In order to check the recognition performance at a flat viewing angle, the camera viewing angle is varying from 0° to 70° by a rotation stage. Figure 17a shows the image at the viewing angle of 70°. The green ‘+’ represent the centers of the CCCTs by the presented positioning method, and the numbers in red represent their ID obtained by our identification method. In Figure 17b, we plot the identification rate as a function of viewing angle. All the coded targets can be identified accurately by our method even at the flat viewing angle of 70°. Therefore, the presented CCCT scheme and related algorithms demonstrate outstanding performances in challenging conditions.

### 4.3. Wing Deformation Measurement Experiment

**Figure 18:**
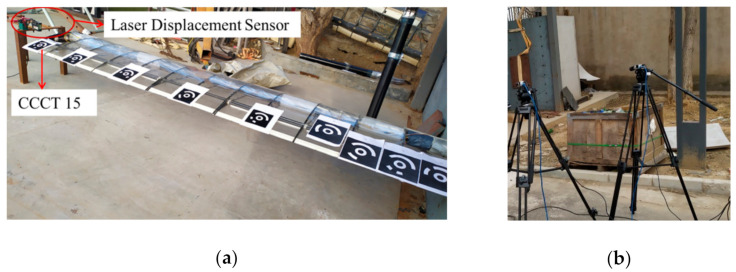
Wing deformation experiment using CCCTs in field work: (**a**) Binocular cameras; (**b**) laser displacement sensor.

For a typical application case mentioned above, we employ the CCCTs to measure the wing deformation of an unmanned aerial vehicle. The length of the wing is about 3.5 m. Nine CCCTs are attached on the wing surface, as shown in Figure 18a. Two Basler digital cameras (Basler acA1300-200 um with a global shutter) both have the resolutions of 1280 × 1024 pixels and are used as image collection equipment, as shown in Figure 18b. Natural sunlight has been adopted for illumination. Before loading the wing, we use Zhang’s method [63] to calibrate the camera parameters of the binocular vision system. Then, a pair of images of the wing is acquired under the unloaded condition. Subsequently, three pairs of images of the wing are taken by the left and right camera when three types of weights (2, 4, and 6 kg) are loaded to the wing tip. Under each loading weight, a KEYENCE G150 laser displacement sensor (LDS) was used to obtain the displacement of CCCT 15 on the wing tip.

**Figure 19:**
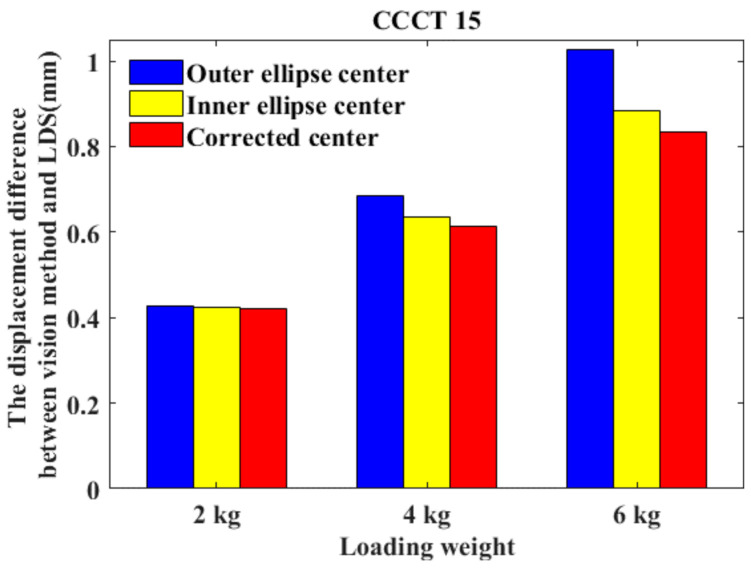
The displacement difference between vision method and laser displacement sensor (LDS) under different loading weights.

Using the proposed algorithms, we can determine the image coordinates of the outer and inner ellipse centers of all the CCCTs in each pair of images and the location of corrected centers. With the camera calibration parameters and the image coordinates, the 3D coordinate of each CCCT can be reconstructed by means of triangulation methods of photogrammetry. The difference between the 3D coordinates of CCCTs under loading the weights and unloaded condition yields the displacement of each CCCT. Under different loading weights, the displacements of CCCT 15 obtained by LDS and vision method based on outer ellipse center, inner ellipse center, and the corrected center are listed in Table 2. It is evident that the displacement obtained by the vision method based on the corrected center is closer to the result of LDS. The displacement difference between the vision method and LDS is shown in Figure 19. Compared with the vision method based on the outer ellipse center, the difference of vision measurement based on the corrected center is reduced by 1.59%, 10.59%, and 18.54% when loading the wing with the weight of 2 kg, 4 kg, and 6 kg. The maximum difference is less than 0.85 mm. When the loading weight is increased, the vision measurement using the proposed eccentricity-error-compensation model has higher accuracy in comparison with the current vision method using only the ellipse center.

**Figure 20:**
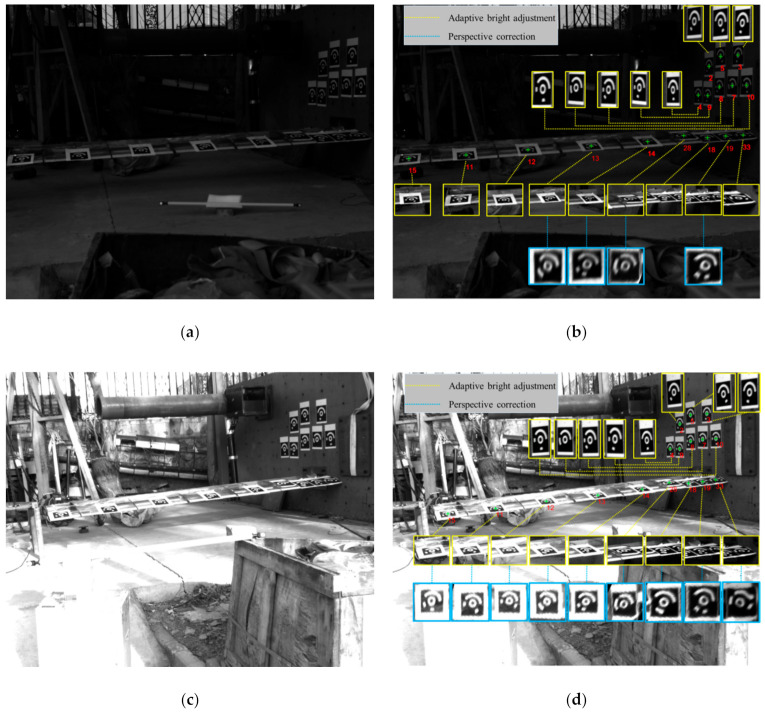
Wing deformation experiment based on CCCTs in field work: (**a**) Underexposure of the image; (**b**) Identification of CCCTs; (**c**) Overexposure; (**d**) Identification of CCCTs.

In addition, Figure 20a shows an example of an underexposed image owing to the significant change in the illumination. Figure 20c gives an example of an overexposed image recorded while having the position of the sun on the unfavorable side of the wing. These two images with difficult illumination conditions have been taken as examples to demonstrate the proposed algorithms. The nine CCCTs attached on the wing have been imaged at a flat viewing angle. Eight CCCTs are attached on the bearing wall for comparison because they are imaged under favorable viewing conditions. The proposed positioning and identification algorithm are applied to process the images. In Figure 20b,d, the numbers in red represent the recognized ID of the CCCTs, and the green “+” are the locations of the corrected centers. It can be seen that all the CCCTs are correctly identified, including a “difficult” one. The local images with yellow borders represent the results subsequent to adaptive bright adjustment. The local images with cerulean borders represent the results subsequent to the perspective correction. We note that the perspective distortions caused by the flat viewing angle are precisely corrected. In real in-flight applications, the above underexposure and overexposure of the recordings could happen owing to flying turns, being into clouds, and the flying direction turning towards the sun. Besides, the problem of perspective distortion at “flat” camera position is unavoidable. The proposed algorithms display a satisfactory effectiveness to cope with these challenges.

## 5. Conclusions

This work proposes a novel concentric circular coded target and its detection algorithm to cope with the problems of coded targets-based vision measurement performed in challenging conditions of poor illumination and flat viewing angle. First, a new design for changing the central positioning dot to a ring has been introduced. This can correct the eccentricity error caused by perspective distortion to improve the positioning accuracy, besides solving the problem of adhesion between the central positioning circle and the circular coded band to improve the recognition rate. Thereafter, we propose a positioning algorithm to precisely locate the true projected center of the CCCT. In this algorithm, the adaptive brightness adjustment is employed to address the problems of poor illumination. Concomitantly, the eccentricity error caused by flat viewing angle is corrected based on a practical error-compensation model. Finally, we present an approach to cope with the decoding failure under a flat viewing angle making CCCTs’ use more robust in challenging environments.

To verify the accuracy and reliability of the proposed positioning algorithm, we have conducted certain simulation experiments under different viewing angles, image resolutions, and image noises. The results indicate that the eccentricity errors of the larger and smaller circles at a large viewing angle of 70° are reduced by 95% and 77% after correction using the proposed method. The front-view image resolution has little or no impact on the locating algorithm. When the image noise is increased, negligible change occurs in the eccentricity errors of outer and inner ellipse centers obtained by fitting, as well as the CCCT corrected center; however, the eccentricity error of the outer ellipse center is five times larger than that of the inner ellipse center, and twenty times that of the corrected center.

To check the proposed identifying method, the experiments under different lighting conditions and viewing angles have been conducted. The recognition rate of all the CCCTs in the field of view is regarded as a criterion for evaluating the recognition performance. The results show that the recognition rate of all the 20 coded targets is still 100% when the average gray value is varying from 12 pixels (underexposure) to 215 pixels (overexposure). All the coded targets can be identified accurately by our method even at the flat viewing angle of 70°.

To further verify the validity, we employ the proposed CCCTs to measure the wing deformation of an unmanned aerial vehicle. The result demonstrates that the error of the vision method based on the corrected center is reduced by up to 18.54% compared with the vision method based on only the ellipse center when the wing is loaded with a weight of 6 kg. Two underexposed and overexposed images captured under the difficult illumination conditions have been taken as examples to demonstrate the identification performance. The identified results demonstrate that the proposed algorithms show appreciable effectiveness to cope with the challenges, for e.g., poor illumination and flat viewing angle.

With the advantages of outstanding positioning and identification performance in challenging conditions of poor illumination and flat viewing angle, we expect an extensive application of the proposed CCCT and its detection algorithms in vision-based industrial measurements.

## Figures and Tables

**Table 1:** The EEs under different image noises.

Gaussian Noise Variance/Pepper Noise Density	The EEs of Outer Ellipse Center by Fitting	The EEs of Inner Ellipse Center by Fitting	The EEs of the CCCT Corrected Center by Our Method
0.005	2.4072/2.4018	0.5238/0.5402	0.1087/0.0826
0.010	2.4072/2.4005	0.5314/0.5157	0.0967/0.1219
0.015	2.4072/2.4170	0.5146/0.5463	0.1186/0.0816
0.020	2.4103/2.3783	0.5165/0.4910	0.1169/0.1575

**Table 2:** The displacement of CCCT 15 on the wing.

Loading Weight	Vision Method Based on Outer Ellipse Center	Vision Method Based on Inner Ellipse Center	Vision Method Based on the Corrected Center	Laser Displacement Sensor
2 kg	10.2315	10.2365	10.2396	10.6599
4 kg	20.5013	20.5491	20.5739	21.1859
6 kg	30.0710	30.2142	30.2637	31.0984

## Data Availability

Data sharing is not applicable to this article.
